# Interaction of physical function, quality of life and depression in Amyotrophic lateral sclerosis: characterization of a large patient cohort

**DOI:** 10.1186/s12883-015-0340-2

**Published:** 2015-05-16

**Authors:** Sonja Körner, Katja Kollewe, Susanne Abdulla, Antonia Zapf, Reinhard Dengler, Susanne Petri

**Affiliations:** Department of Neurology, Medizinische Hochschule Hannover, Carl-Neuberg-Str. 1, 30625 Hannover, Germany; Department of Neurology, Otto von Guericke University, Magdeburg, Germany; Department of Medical Statistics, University Göttingen, Göttingen, Germany; Center for Systems Neuroscience (ZSN), Hannover, Germany

**Keywords:** Amyotrophic lateral sclerosis, Depression, Quality of life, Palliative care

## Abstract

**Background:**

Due to lack of any curative therapy for ALS, symptomatic treatment and maintenance of quality of life (QoL) is very important. We aimed to characterize the affected domains of QoL in ALS patients and to identify factors which are associated with reduced QoL and increased depression.

**Methods:**

159 ALS patients answered standardized questionnaires (Beck Depression Inventory-II, SF-36 Health Survey questionnaire, revised ALS functional rating scale). Multiple regression analysis was used to identify correlations between clinical features of ALS patients and depression/QoL scores. In addition, QoL data from ALS patients were compared to age-matched reference values representing the German normal population.

**Results:**

QoL of ALS patients was reduced in nearly all SF-36-categories. Progression of physical impairment was positively correlated with depression but reduced QoL scores only in items directly related to physical function. However, QoL was considerably influenced by depression, independently from physical impairment. Regarding distinct patient characteristics one of the most interesting findings was that increasing age was correlated with significantly worse QoL results regarding social functioning.

**Conclusions:**

Depressive symptoms had a strong influence on QoL, hence their detection and treatment is of particular importance. Different domains of QoL are differently affected in subgroups of ALS patients. Being aware of these differences can be valuable for both ALS professional and family caregivers and physicians.

**Electronic supplementary material:**

The online version of this article (doi:10.1186/s12883-015-0340-2) contains supplementary material, which is available to authorized users.

## Background

Amyotrophic lateral sclerosis (ALS) is the most common motor neuron disease in adults. Degeneration of upper and lower motor neurons leads to rapidly progressive paralysis of skeletal muscles, thereby also affecting respiratory, speech and swallowing functions. Therefore ALS patients are confronted with progressive physical impairment and loss of communication [1]. Despite intensive research efforts, curative therapy is lacking so far. Thus palliative care of patients suffering from this incurable and terminal disease with the aim to alleviate disease symptoms as much as possible is of high importance. Clinical care and management has become a topic of increasing research interest [2–4]. Improvement of quality of life (QoL) is most relevant as patients with psychological distress have a greater risk of mortality [5].

Many studies have reported surprisingly good QoL in ALS patients despite the dramatic and rapidly progressive physical impairment [2–4, 6–8]. The interaction of disability and depression respectively QoL in ALS has been controversially discussed [2–4, 6, 7, 9, 10]. Does physical disability automatically lead to development of depression and reduced quality of life? Or are patients able to adapt to the new situation and to replace activities no longer possible due to disease-related physical impairment (profession, hobbies) by other similarly satisfying interests? And if the latter statement is true, does it also apply to ALS patients where disability rapidly deteriorates with disease progression and therefore constantly requires new adjustments? Differential results of previous studies regarding this issue are possibly due to low sample sizes and to the use of different tools for assessment of QoL and depressive symptoms. Moreover, evaluation of these items is especially difficult in ALS patients as they are restricted in many activities due to disease-related physical limitations. Hence QoL in ALS patients must be determined in different areas of life. Until now, it is still unclear to what extent progressive physical limitations are necessarily associated with increasing depression and decreasing QoL and which other factors are important.

In our study in a large cohort of 159 ALS patients we intended to characterize the impact of several factors (physical impairment, disease duration, gender, disease onset and age) on depressive symptoms and QoL in ALS patients and to specify the relationship between physical impairment, depression and QoL. We further aimed to elucidate which aspects of QoL are affected in ALS patients and whether there are differences related to the disease phenotype. We aimed to provide more systematic and detailed evidence for the relevance of ALS-related limitations in physical and social functions. This knowledge should contribute to more specific therapy and care, adjusted to the individual situation of a patient.

## Methods

One hundred fifty nine ALS patients from the ALS outpatient clinic at Hannover Medical School participated in the survey. The study has been approved by the ethical committee of Hannover Medical School and all subjects gave informed consent to the participation. The study was performed in accordance with the Declaration of Helsinki principles. All patients had met the revised El Escorial criteria for probable or definite ALS [11] and did not have clinical signs of frontotemporal dementia, while specific tests to detect minor cognitive/frontotemporal abnormalities were not routinely performed. Patients filled in three standardized questionnaires (Beck Depression Inventory - II (BDI), SF-36 Health Survey questionnaire (SF-36) and revised ALS Functional Rating Scale (ALSFRS-R)).

The ALSFRS-R is a well-established and widely used score for the functional status of patients with ALS. It is based on 12 items, each rated on a 0–4 point scale. The rate of functional disability ranges from 0 (maximum disability) to 48 (normal) points. Three items of the ALSFRS-R assess bulbar involvement (speech, salivation, swallowing), which therefore can be rated from 0 (maximum bulbar involvement) to 12 (no bulbar involvement) [12]. Further six items of the ALSFRS-R address motor function of upper and lower limbs (handwriting, cutting food, dressing and hygiene, walking, turning in bed, climbing stairs). The last three items estimate involvement of respiratory function (dyspnoea, orthopnoea, respiratory insufficiency).

The BDI is a 21-question multiple-choice self-report inventory, commonly used for quantifying levels of depression. Each of the 21 items is scored on a scale from 0 (symptom not present) to 3 (symptom very intense) leading to an overall-range of 63. The cutoffs used are 0–8: no depressive symptoms, 9–13: minimal depressive symptoms, 14–19: mild depressive symptoms, 20–28: moderate depressive symptoms, 29–63 severe depressive symptoms [13].

The SF-36 questionnaire is a multi-purpose, short-form health survey with 36 questions. It is a self-administered QoL scoring system that includes eight independent scales: 1. Physical functioning (limitations in physical activities), 2. Physical role (limitations in usual role activities because of physical health problems), 3. Bodily pain, 4. General health perception, 5. Vitality (energy and fatigue), 6. Social functioning (limitations in social activities because of physical or emotional problems), 7. Emotional role (limitations in usual role activities because of emotional problems), 8. Mental health (psychological distress and well-being) [14]. Each of the question responses relate to a different pre-coded numeric value. The sums of the questions in their section form raw scores for each of the eight scales. The raw scores are translated into one from 0 to 100, with 0 representing a very low level of QoL in that item and 100 representing a very positive response. The SF–36 was chosen as it is short, easy to complete and covers areas that appear appropriate to patients with ALS, such as ability to perform work and daily activities, physical functioning and social functioning. The questionnaire is widely used and has undergone a substantial amount of testing for reliability, validity and responsiveness; it has further been proven to be an adequate instrument for ALS patients [15, 16].

The SF-36 was standardized in a German norm population (*n* = 2914) [17]. Age-specific reference values generated in this population have already been applied in other studies for comparison of QoL of patients with different diseases [18, 19] and were also used in the present study to compare QoL of ALS patients with the German general population. If the reference value was outside the 95 % confidence interval regarding the ALS study population, we concluded a significant difference.

The ALS patient group was characterized regarding gender, onset, age, disease duration and disease severity (see Table 1). For variables with an asymptotic normal distribution mean and standard deviation (SD) are reported, for variables without asymptotic normal distribution median, minimum and maximum are reported in addition. To identify correlations between disease severity, age and disease duration with the extent of depression (BDI) and different aspects of QoL (SF-36), we used linear regression analysis. We first performed a simple regression analysis. Afterwards all variables which tended to significance (*p*-value < 0.2) were included in a multiple regression model. In a last step we used backward selection (with p > 0.05 as exclusion criterion) to determine the final model. All results including regression coefficent ß, confidence interval for ß and *p*-values for each of these calculations are listed in Additional file 1: Table S1. A summary of the results is provided in Table 3. Statistical analyses were performed using SPSS V. 19 (SPSS, Chicago, IL) software, a *p*-value of <0.05 was considered significant.

**Table 1:** Characteristics of the ALS patient cohort

Characteristics of patient cohort	ALS patients
Gender (male : female), *n* = 159	92: 67 (58 % : 42 %)
Onset (spinal : bulbar), *n* = 155	113: 42 (73 % : 27 %)
Age, *n* = 159	
mean (SD)	60.7 years (SD 11.1)
Disease duration, *n* = 145	
mean (SD)	40.2 months (SD 43.1)
median (min - max)	24 months (1 – 234)
ALSFRS-R, *n* = 155	
mean (SD)	28.46 (SD 10.9)
ALSFRS-R bulbar, *n* = 155	
mean (SD)	7.68 (SD 3.8)
median (min - max)	9 (0–12)

ALSFRS-R: Amyotrophic Lateral Sclerosis Functional Rating Scale – Revised

SD: standard deviation

**Table 2:** Association of different patient characteristics with depression (BDI) and quality of life (SF-36) scores

	BDI	Physical functioning	Physical role	Bodily pain	General health	Vitality	Social functioning	Emotional role	Mental health
**ALSFRS-R** ↓	↑ **(p < 0.001)**	↓ **(p < 0.001)**	0.092	-	0.731	↓ **(p < 0.01)**	0.175	0.774	0.052
**ALSFRS-R bulbar** ↓	0.345	-	-	↑ **(p < 0.01)**	-	0.116	0.864	-	0.555
**Disease duration**	-	-	-	0.113	-	-	-	-	-
**Gender**	-	-	-	-	-	-	-	-	-
**Onset**	-	**Sig.** ↓ **for spinal onset (p < 0.01)**	**Sig.** ↓ **for spinal onset (p < 0.001)**	0.743	-	-	-	-	-
**Age** ↑	0.126	0.072	-	-	0.058	-	↓ **(p < 0.05)**	-	0.292
**BDI** ↑	n.a.	0.132	↓ **(p < 0.001)**	↓ **(p < 0.05)**	↓ **(p < 0.001)**	↓ **(p < 0.001)**	↓ **(p < 0.001)**	↓ **(p < 0.001)**	↓ **(p < 0.001)**

-: *p*-value >0.2 in the simple regression, variable not included in the final multiple regression model

n.a.: not applicable

ALSFRS-R: Amyotrophic Lateral Sclerosis Functional Rating Scale – Revised

BDI: Beck Depression Inventory

**Table 3:** Prevalence of different degrees of depression according to the BDI scores

Degree of depression	BDI complete (range 0–63)		BDI without somatic items (range 0–45)	
	Range	Prevalence (%)	Range	Prevalence (%)
No depressive symptoms	BDI 0-8	19 %	BDI 0-5	30 %
Minimal depressive symptoms	BDI 9-13	24 %	BDI 6-9	29 %
Mild depressive symptoms	BDI 14-19	26 %	BDI 10-13	16 %
Moderate depressive symptoms	BDI 20-28	19 %	BDI 14-20	18 %
Severe depressive symptoms	BDI 29-63	12 %	BDI 21-45	7 %

BDI: Beck Depression Inventory

## Results

We analysed data from 159 ALS patients (92 male and 67 female) with a mean age of 60.69 years (standard deviation of 11.12 years) and a median disease duration of 24 months (range: 1 month to 19.5 years). Further characteristics of the cohort are listed in Table 1.

As revealed by BDI scores, depressive symptoms were frequent in ALS. Using the regular BDI score, 30 % of the patients suffered from moderate or severe depressive symptoms (Table 2). Since the BDI is often criticized for overestimating depression in physically handicapped persons because of its somatic items, we also calculated the prevalence of depression after removal of the somatic items (regarding ability to work, sleep, exhaustion, appetite, weight loss and concern about health) in new approximate ranges. This analysis showed indeed a lower prevalence in the category “mild depressive symptoms”, while the frequency of moderate or severe depressive symptoms was still 25 % (Table 2).

**Fig. 1:**
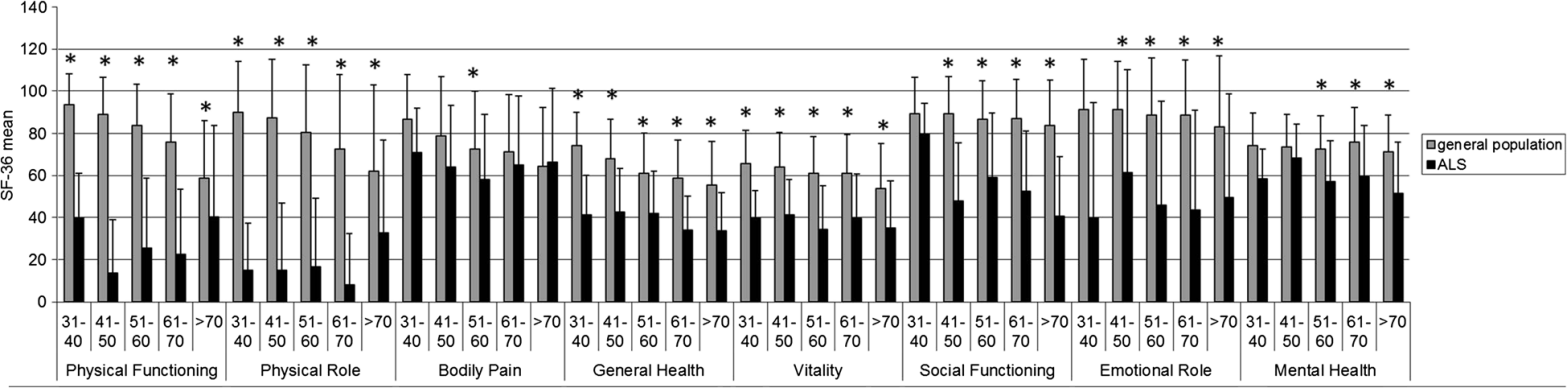
Comparison of quality of life (QoL) in ALS patients to age-group specific reference values of the German general population. QoL is declined in ALS patients in nearly every scale. Limitations in the field “mental health” only become apparent in older patients, and in the subscale “bodily pain” QoL of ALS patients barely differs from the general population. As in the scales “Physical Functioning”, “Physical Role”, “Bodily Pain” and “Emotional Role” a normal distribution cannot be assumed the comparison in these fields can only be rated descriptive. Sample sizes: 31–40 years 5 patients, 41–50 years 22 patients, 51–60 years 53 patients, 61–70 years 42 patients, >70 years 37 patients

Compared to the German general population in age-matched groups, QoL was significantly reduced in our ALS patient cohort in all categories represented by the SF-36 subscales except for the item “Bodily pain” where ALS patients only marginally differed from the general population. Differences related to “mental health” seemed to be only relevant in older patients (older than 51 years) (Fig. 1).

**Fig. 2:**
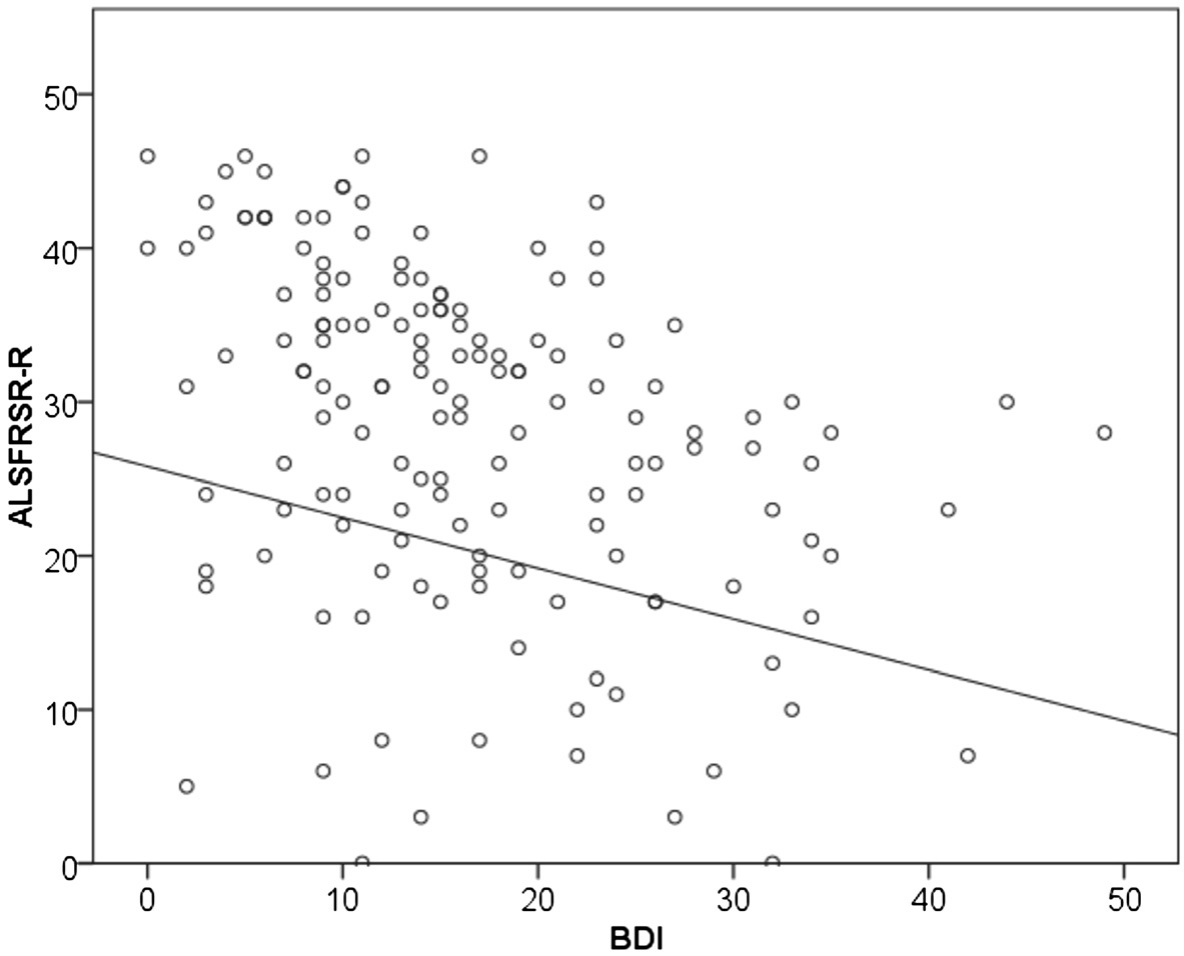
Linear regression analysis showed a significant negative correlation between ALSFRS-R and BDI. Advanced physical impairment (lower ALSFRS-R) was correlated with increasing depression (higher BDI scores)

Linear regression analysis was performed to identify impact factors on depression and QoL. The results of the final model after backward selection are displayed in Table 3. (All results including the results of the simple regression models are summarized in Additional file 1: Table S1). Advanced physical impairment (lower ALSFRS-R) was correlated with increasing depression (higher BDI scores) (Fig. 2). The multiple regression analysis showed that the extent of physical impairment also had an independent impact on the QoL (SF-36) scales related to physical functioning and vitality, regardless of the severity of depression, while the other six categories were not affected (Table 3). The level of depression, on the other hand, independent of the ALSFRS-R score, significantly influenced the majority of QoL categories, including general health, vitality, social functioning, emotional role and mental health (Table 3). That was also the case when we used the BDI score without somatic items, except for the QoL categories physical role and bodily pain (Additional file 2: Table S2).

Patients with a lower ALSFRS-R bulbar score had significantly less bodily pain. Pain in ALS is typically caused by muscle atrophy and subsequent increase in sensitivity of bones and joints, limited mobility as well as muscle cramps and spasticity [20]. Patients with predominant bulbar involvement are to a lesser extent restricted in their motility and therefore probably suffer less from pain due to immobility. As, on the other hand, patients with spinal onset develop weakness and atrophy of limb muscles as initial symptoms, they had significantly worse QoL scores in the categories “physical functioning and role” (Table 3).

Higher age was correlated with lower SF-36 scores regarding social functioning (Table 3).

Gender had no influence on depression or QoL.

Surprisingly, disease duration had no influence on depression and QoL either, although longer disease duration was correlated with lower ALSFRS-R scores (p < 0.01) and similar correlations as for the ALSFRS-R could have been expected.

## Discussion

### Quality of life in ALS patients – comparison with general population-derived reference values

In our study, QoL was significantly reduced in ALS patients compared to reference values from the general population [17] in nearly all subscales. This reduction of QoL in ALS patients has been described before in one study which also used the SF-36 questionnaire [15]. Other studies, however, resulted in contrary results: a direct comparison of ALS patients with healthy control persons revealed no differences in QoL [4]; in a study by Kübler et al., 80 % of ALS patients rated their QoL as satisfactory or good [2]. QoL includes many domains of experience which are differently affected in ALS and differently rated by ALS patients. One explanation for these controversial results might be the differential emphasis of different tools to assess QoL. There is an ongoing discussion whether so called health related questionnaires as the SF-36 are generally suitable in ALS patients. They are often criticized for focusing mainly on functional status and not being able to accurately reflect the patients’ individual QoL [21–23]. Those studies suggest to rather use more individualized questionnaires as e.g., the SEIQoL-DW (Schedule for the Evaluation of Individual Quality of Life Direct Weight) which allows patients to focus on fields of their life which they consider relevant and which are not influenced by physical impairment. These questionnaires indeed highlight QoL more from the patients’ perspective. But they also have disadvantages: In our clinical experience, patients always name areas and activities they can still perform without difficulties, which results in relatively high QoL scores. These tests therefore demonstrate that ALS patients are able to shift their priorities and thereby maintain their QoL to a certain degree. We think, however, that they are at risk of overlooking categories of QoL which are affected in ALS patients. One could therefore wrongly assume that there is nothing to improve in the symptomatic treatment/care of ALS patients. General health status questionnaires such as the SF-36, on the other hand, separately assess physical, mental, social and emotional factors. They have the disadvantage that some subscales depend on physical abilities which we clearly accept as a limitation of our study. However, in our analysis, ALS patients rated their QoL worse not only in the physical categories but also in nearly all other dimensions. There are already few studies which used several questionnaires from both categories [21–23]. Nevertheless further research is required. It would be interesting to combine different questionnaires with objective and subjective assessment of QoL, using disease-specific and more general questionnaires in large ALS populations, to give the most comprehensive estimation of well-being of ALS patients.

In fact, in the present study the most obvious difference in QoL between ALS patients and general population was observed regarding the physical domains (physical functioning and physical role). This result was to be expected but should not be considered unchangeable. In our experience, patients often hesitate to use walking frames or wheel chairs and rather accept immobility which apparently has a considerable negative impact on QoL. Based on our findings, it should be at least attempted to achieve maximum improvement in the physical domain by use of assistive technologies. Higher autonomy and mobility might subsequently even improve QoL in other categories, namely regarding social functioning which was also significantly reduced in ALS patients. An early prescription of assistive technologies by the treating neurologist or family practitioner as well as sufficient guidance by physical therapists and medical supply stores seems to be essential. Future studies will be necessary to specifically analyse the effect of early and comprehensive equipment with assistive devices capable to improve mobility and communication on QoL of ALS patients.

The scores in the category “bodily pain” were similar in the ALS cohort compared to the reference values, as opposed to all other SF-36 scales. A prevalence of pain in ALS patients up to 50 % has been described [24]. In another study which specifically addressed this issue, the median physical pain score reported by ALS patients was 2 on a six point scale, but 20 % of patients rated their pain as ≥4 [25], indicating that even though pain was not a common problem in our ALS cohort, a subset of ALS patients does suffer from severe pain.

### Factors associated with higher levels of depression and decreased quality of life in ALS patients

Severity of depressive symptoms in our cohort was positively correlated to the extent of physical impairment. This has been controversially described in previous studies: Some authors support a relationship between depression and disease stage [2, 9, 10, 26] while others do not [4, 27]. However, one must differentiate between advanced physical impairment (i.e., deterioration of ALSFRS-R) and just longer disease duration. Despite progressive physical impairment during disease duration and a positive correlation between the severity of depression and physical impairment, there was no significant correlation between depression and disease duration in our cohort. This finding is in line with a previous study which found even a negative correlation between depressive symptoms and time since diagnosis [2]. With longer disease duration patients can develop strategies to cope with the diagnosis and the physical impairment; hence depressive symptoms are less frequent or at least not progressive with longer disease duration despite increasing disability.

According to the literature, physical impairment does not influence QoL [4, 7, 23, 28]. We found that in fact physical impairment (i.e., low ALSFRS-R scores) only had a significant impact on the physical functioning and vitality scale of the SF-36 but did not influence other aspects. Severity of depression as measured by BDI, on the contrary, had a substantial impact on QoL on several scales including general health, vitality, mental health as well as social and emotional well-being. This impact was not modified by the extent of physical impairment. Our results are in line with the literature as it has been described previously that depression and QoL are highly negatively related [29]. In ALS patients, fatigue and depression were previously found to be associated with poor QoL [23, 30, 31].

Data on prevalence of depression in ALS in different studies depend on the tests they used, but there is some evidence that depressive symptoms are frequent [2, 26, 30, 32–34]. In the present study over 30 % of patients had moderate or severe depressive symptoms. Although the BDI as well as other depression scores include somatic markers of depression and therefore can overestimate the prevalence of depression in ALS patients [8, 20, 35–37], it has been used in ALS patients and a high reliability of the BDI in assessing self-reported depressive symptoms has been shown [10, 38]. Moreover, the prevalence of moderate or severe depressive symptoms in our ALS population was only slightly lower when we removed the somatic items from the BDI. Hence a significant proportion of ALS patients seem to suffer from depression. It can neither be recommended to use the BDI as a depression screening instrument nor to treat ALS patients with antidepressants based on their BDI score. One must, however, be aware of the frequency of depressive symptoms in ALS patients and pharmacological treatment as well as psychological support should be made available if necessary. The significant correlation between depression and QoL clearly suggests that efficient treatment of depression could contribute to improve QoL. Depressive symptoms should therefore not be accepted as an inevitable consequence of an incurable disease [38].

Regarding QoL in elderly ALS patients, we observed a significant negative correlation of age with the domain social functioning. This means that increasing age maybe is another factor which adds to the limitation of social contacts. Health professionals as well as family members should have that in mind and specifically address this issue in older ALS patients.

## Conclusion

Our study answers the questions raised in the background sections as follows: QoL was considerably reduced in ALS patients, especially regarding (but not restricted to) physical domains. Physical impairment promotes depressive symptoms but does not necessarily lead to reduced QoL. However, depressive symptoms are frequently observed in ALS and can have a serious negative effect on QoL. Identification of other potentially treatable factors which promote development of depression in ALS patients could therefore be useful.

One can conclude that, despite the lack of curative therapy, ALS carers (including specialized physicians as well as nursing and familial caregivers) can substantially contribute to maintenance of QoL at the highest possible level. QoL and depressive symptoms should be regularly evaluated and special requirements of patients with different ALS phenotypes should be kept in mind.

## Recommendations

1. Advanced physical impairment was correlated with higher degrees of depression. Hence, assistive devices for improved mobility and autonomy should be provided by clinicians specialized in ALS and sound instructions by physical therapists should ensure proper use by patients.
2. QoL was much more influenced by depression than by physical impairment. Timely diagnosis and pharmacological treatment of depressive symptoms is therefore crucial in ALS and could improve QoL.
3. Low-threshold psychotherapeutic support should be available for ALS patients.
4. In elderly patients, health professionals and family caregivers should particularly focus on social functioning as reduction in QoL in this category is age-dependent.
5. Implementation of point 1–4 requires involvement of neurologists specialized in ALS, qualified physical therapists and instructors in medical supply stores, psychiatrists and/or psychotherapists as well as sensitive professional or familial nursing staff, thereby highlighting the importance of a multidisciplinary team for the treatment of ALS-patients.

## Additional files

Additional file 1: Table S1. Detailed results of regression analysis. Additional file 2: Table S2. Association of different patient characteristics with depression (BDI without somatic items) and quality of life (SF-36) scores.

## Abbreviations

ALS: Amyotrophic lateral sclerosis
ALSFRS-R: ALS functional rating scale – revised version
BDI: Beck depression inventory
QoL: Quality of life
SF-36: Short form 36 health survey

## References

[1] Wijesekera LC, Leigh PN (2009). Amyotrophic lateral sclerosis. Orphanet J Rare Dis.

[2] Kubler A, Winter S, Ludolph AC, Hautzinger M, Birbaumer N (2005). Severity of depressive symptoms and quality of life in patients with amyotrophic lateral sclerosis. Neurorehabil Neural Repair.

[3] Kiebert GM, Green C, Murphy C, Mitchell JD, O’Brien M, Burrell A (2001). Patients’ health-related quality of life and utilities associated with different stages of amyotrophic lateral sclerosis. J Neurol Sci.

[4] Lule D, Hacker S, Ludolph A, Birbaumer N, Kubler A (2008). Depression and quality of life in patients with amyotrophic lateral sclerosis. Dtsch Arztebl Int.

[5] McDonald ER, Wiedenfeld SA, Hillel A, Carpenter CL, Walter RA (1994). Survival in amyotrophic lateral sclerosis. The role of psychological factors. Arch Neurol.

[6] Rabkin JG, Albert SM, Del Bene ML, O’Sullivan I, Tider T, Rowland LP (2005). Prevalence of depressive disorders and change over time in late-stage ALS. Neurology.

[7] Robbins RA, Simmons Z, Bremer BA, Walsh SM, Fischer S (2001). Quality of life in ALS is maintained as physical function declines. Neurology.

[8] Pagnini F, Manzoni GM, Tagliaferri A, Gibbons CJ. Depression and disease progression in amyotrophic lateral sclerosis: A comprehensive meta-regression analysis. J Health Psychol. 2014. 10.1177/1359105314530453 24764286

[9] Hunter MD, Robinson IC, Neilson S (1993). The functional and psychological status of patients with amyotrophic lateral sclerosis: some implications for rehabilitation. Disabil Rehabil.

[10] Oh H, Sin MK, Schepp KG, Choi-Kwon S (2012). Depressive symptoms and functional impairment among amyotrophic lateral sclerosis patients in South Korea. Rehabil Nurs.

[11] Brooks BR, Miller RG, Swash M, Munsat TL (2000). El Escorial revisited: revised criteria for the diagnosis of amyotrophic lateral sclerosis. Amyotroph Lateral Scler Other Motor Neuron Disord.

[12] Cedarbaum JM, Stambler N, Malta E, Fuller C, Hilt D, Thurmond B (1999). The ALSFRS-R: a revised ALS functional rating scale that incorporates assessments of respiratory function. BDNF ALS Study Group (Phase III). J Neurol Sci.

[13] Beck AT, Steer RA, Ball R, Ranieri W (1996). Comparison of Beck Depression Inventories -IA and -II in psychiatric outpatients. J Pers Assess.

[14] Ware JE, Sherbourne CD (1992). The MOS 36-item short-form health survey (SF-36). I. Conceptual framework and item selection. Med Care.

[15] Jenkinson C, Fitzpatrick R, Swash M, Peto V (2000). The ALS Health Profile Study: quality of life of amyotrophic lateral sclerosis patients and carers in Europe. J Neurol.

[16] Jenkinson C, Hobart J, Chandola T, Fitzpatrick R, Peto V, Swash M (2002). Use of the short form health survey (SF-36) in patients with amyotrophic lateral sclerosis: tests of data quality, score reliability, response rate and scaling assumptions. J Neurol.

[17] Bullinger M, Kirchberger I (1998). SF-36 Fragebogen zum Gesundheitszustand.

[18] Jäger S, Jagla M, Morefeld M, Türk T, Witzke O, Reimer J (2009). Gesundheitsbezogene Lebensqualität bei Patienten nach Nierentransplantation - Lässt sich die Skalenstruktur des SF-36 replizieren?. Diagnostica.

[19] Reimer J, Rensing A, Haasen C, Philipp T, Pietruck F, Franke GH (2006). The impact of living-related kidney transplantation on the donor’s life. Transplantation.

[20] Blackhall LJ (2012). Amyotrophic lateral sclerosis and palliative care: where we are, and the road ahead. Muscle Nerve.

[21] Neudert C, Wasner M, Borasio GD (2001). Patients’ assessment of quality of life instruments: a randomised study of SIP, SF-36 and SEIQoL-DW in patients with amyotrophic lateral sclerosis. J Neurol Sci.

[22] Neudert C, Wasner M, Borasio GD (2004). Individual quality of life is not correlated with health-related quality of life or physical function in patients with amyotrophic lateral sclerosis. J Palliat Med.

[23] Chio A, Gauthier A, Montuschi A, Calvo A, Di VN, Ghiglione P (2004). A cross sectional study on determinants of quality of life in ALS. J Neurol Neurosurg Psychiatry.

[24] Pagnini F, Lunetta C, Banfi P, Rossi G, Fossati F, Marconi A (2012). Pain in Amyotrophic Lateral Sclerosis: a psychological perspective. Neurol Sci.

[25] Ganzini L, Johnston WS, Hoffman WF (1999). Correlates of suffering in amyotrophic lateral sclerosis. Neurology.

[26] Hogg KE, Goldstein LH, Leigh PN (1994). The psychological impact of motor neurone disease. Psychol Med.

[27] Rabkin JG, Wagner GJ, Del BM (2000). Resilience and distress among amyotrophic lateral sclerosis patients and caregivers. Psychosom Med.

[28] Simmons Z, Bremer BA, Robbins RA, Walsh SM, Fischer S (2000). Quality of life in ALS depends on factors other than strength and physical function. Neurology.

[29] Badger TA (2001). Depression, Psychological Resources, and Health-Related Quality of Life in Older Adults 75 and Above. J Clin Geropsychol.

[30] Lou JS, Reeves A, Benice T, Sexton G (2003). Fatigue and depression are associated with poor quality of life in ALS. Neurology.

[31] Gibbons C, Thornton E, Ealing J, Shaw P, Talbot K, Tennant A (2013). The impact of fatigue and psychosocial variables on quality of life for patients with motor neuron disease. Amyotroph Lateral Scler Frontotemporal Degener.

[32] Moore MJ, Moore PB, Shaw PJ (1998). Mood disturbances in motor neurone disease. J Neurol Sci.

[33] Korner S, Kollewe K, Ilsemann J, Muller-Heine A, Dengler R, Krampfl K (2012). Prevalence and prognostic impact of comorbidities in Amyotrophic Lateral Sclerosis. Eur J Neurol.

[34] Wicks P, Abrahams S, Masi D, Hejda-Forde S, Leigh PN, Goldstein LH (2007). Prevalence of depression in a 12-month consecutive sample of patients with ALS. Eur J Neurol.

[35] Taylor L, Wicks P, Leigh PN, Goldstein LH (2010). Prevalence of depression in amyotrophic lateral sclerosis and other motor disorders. Eur J Neurol.

[36] Ferentinos P, Paparrigopoulos T, Rentzos M, Zouvelou V, Alexakis T, Evdokimidis I (2011). Prevalence of major depression in ALS: comparison of a semi-structured interview and four self-report measures. Amyotroph Lateral Scler.

[37] Gibbons CJ, Mills RJ, Thornton EW, Ealing J, Mitchell JD, Shaw PJ (2011). Rasch analysis of the hospital anxiety and depression scale (HADS) for use in motor neurone disease. Health Qual Life Outcomes.

[38] Simmons Z (2013). Rehabilitation of motor neuron disease. Handb Clin Neurol.

