# Supplementary material for: Interaction of physical function, quality of life and depression in Amyotrophic lateral sclerosis: characterization of a large patient cohort
Source: BMC Neurol. 2015 May 16;15:84. doi: 10.1186/s12883-015-0340-2 (PMC4493831; doi:10.1186/s12883-015-0340-2)
Supplement: Additional file 1: Table S1. — Detailed results of regression analysis. [file 12883_2015_340_MOESM1_ESM.docx]

**Additional file 1: Table S1**

Detailed results of regression analysis

| **Dependent Variable: Physical functioning** | | | |
| --- | --- | --- | --- |
| **Simple regression** | | | |
| **Predictor Variable** | **Regressioncoeff. β** | **95% CI for β** | **p-value** |
| ALSFRS-R total | 1.258 | 0.775 ; 1.74 | <0.001 |
| ALSFRS-R bulbar | 0.669 | -0.791 ; 2.128 | 0.367 |
| Disease duration | -0.089 | -0.227 ; 0.049 | 0.206 |
| Gender | -7.121 | -18.295 ; 4.052 | 0.21 |
| Onset | 14.754 | 2.344 ; 27.164 | <0.05 |
| Age | 0.505 | -0.001 ; 1.012 | 0.05 |
| BDI | -0.89 | -1.466 ; -0.315 | <0.01 |
| **Multiple regression** |  |  |  |
| ALSFRS-R total | 1.19 | 0.675 ; 1.706 | <0.001 |
| Onset | 17.898 | 6.358 ; 29.437 | <0.01 |
| Age | 0.485 | 0.015 ; 0.954 | <0.05 |
| BDI | -0.514 | -1.084 ; 0.055 | 0.076 |
| **Final model after backwards selection** |  |  |  |
| **ALSFRS-R total** | **1.365** | **0.883 ; 1.847** | **<0.001** |
| **Onset** | **20.661** | **9.181 ; 32.141** | **<0.01** |
| Age | 0.43 | -0.04 ; 0.899 | 0.072 |
| BDI | -0.514 | -1.084 ; 0.055 | 0.076 |

| **Dependent Variable: Physical role** | | | |
| --- | --- | --- | --- |
| **Simple regression** | | | |
| **Predictor Variable** | **Regressioncoeff. β** | **95% CI for β** | **p-value** |
| ALSFRS-R total | 0.682 | 0.17 ; 1.194 | <0.01 |
| ALSFRS-R bulbar | -0.962 | -2.446 ; 0.522 | 0.202 |
| Disease duration | -0.049 | -0.185 ; 0.087 | 0.481 |
| Gender | 5.75 | -5.561 ; 17.061 | 0.317 |
| Onset | 21.15 | 9.451 ; 32.849 | <0.001 |
| Age | 0.308 | -0.197 ; 0.813 | 0.23 |
| BDI | -0.916 | -1.484 ; -0.349 | <0.01 |
| **Multiple regression** |  |  |  |
| ALSFRS-R total | 0.459 | -0.075 ; 0.993 | 0.092 |
| Onset | 23.223 | 11.658 ; 34.788 | <0.001 |
| BDI | -0.8 | -1.39 ; -0.211 | <0.01 |
| **Final model after backwards selection** |  |  |  |
| ALSFRS-R total | 0.459 | -0.075 ; 0.993 | 0.092 |
| **Onset** | **21.656** | **10.156 ; 33.156** | **<0.001** |
| **BDI** | **-1.007** | **-1.549 ; -0.465** | **<0.001** |

| **Dependent Variable: Bodily Pain** | | | |
| --- | --- | --- | --- |
| **Simple regression** | | | |
| **Predictor Variable** | **Regressioncoeff. β** | **95% CI for β** | **p-value** |
| ALSFRS-R total | 0.217 | -0.252 ; 0.687 | 0.361 |
| ALSFRS-R bulbar | -1.32 | -2.647 ; 0.007 | 0.051 |
| Disease duration | -0.111 | -0.234 ; 0.012 | 0.076 |
| Gender | 2.244 | -7.974 ; 12.463 | 0.665 |
| Onset | 11.335 | 0.006 ; 22.664 | 0.05 |
| Age | 0.021 | -0.439 ; 0.48 | 0.929 |
| BDI | -0.568 | -1.092 ; -0.043 | <0.05 |
| **Multiple regression** |  |  |  |
| ALSFRS-R bulbar | -1.869 | -3.585 ; -0.153 | <0.05 |
| Disease duration | -0.096 | -0.219 ; 0.026 | 0.121 |
| Onset | 2.258 | -11.309 ; 15.825 | 0.743 |
| BDI | -0.711 | -1.304 ; -0.117 | <0.05 |
| **Final model after backwards selection** |  |  |  |
| **ALSFRS-R bulbar** | **-2.023** | **-3.476 ; -0.571** | **<0.01** |
| Disease duration | -0.098 | -0.219 ; 0.024 | 0.113 |
| Onset | 2.258 | -11.309 ; 15.825 | 0.743 |
| **BDI** | **-0.723** | **-1.311 ; -0.134** | **<0.05** |

| **Dependent Variable: General Health** | | | |
| --- | --- | --- | --- |
| **Simple regression** | | | |
| **Predictor Variable** | **Regressioncoeff. β** | **95% CI for β** | **p-value** |
| ALSFRS-R total | 0.259 | -0.27 ; 0.546 | 0.076 |
| ALSFRS-R bulbar | 0.182 | -0.633 ; 0.996 | 0.66 |
| Disease duration | 0.001 | -0.073 ; 0.075 | 0.978 |
| Gender | 0.879 | -5.314 ; 7.073 | 0.779 |
| Onset | 2.079 | -4.856 ; 9.013 | 0.555 |
| Age | -0.353 | -0.628 ; -0.078 | <0.05 |
| BDI | -0.888 | -1.176 ; -0.601 | <0.001 |
| **Multiple regression** |  |  |  |
| ALSFRS-R total | -0.049 | -0.329 ; 0.231 | 0.731 |
| Age | -0.248 | -0.504 ; 0.009 | 0.058 |
| BDI | -0.872 | -1.187 ; -0.557 | <0.001 |
| **Final model after backwards selection** |  |  |  |
| ALSFRS-R total | -0.049 | -0.329 ; 0.231 | 0.731 |
| Age | -0.247 | -0.503 ; 0.008 | 0.058 |
| **BDI** | **-0.89** | **-1.181 ; -0.598** | **<0.001** |

| **Dependent Variable: Vitality** | | | |
| --- | --- | --- | --- |
| **Simple regression** | | | |
| **Predictor Variable** | **Regressioncoeff. β** | **95% CI for β** | **p-value** |
| ALSFRS-R total | 0.676 | 0.389 ; 0.963 | <0.001 |
| ALSFRS-R bulbar | 0.762 | -0.09 ; 1.615 | 0.079 |
| Disease duration | 0.016 | -0.063 ; 0.095 | 0.683 |
| Gender | 3.133 | -3.475 ; 9.741 | 0.35 |
| Onset | -1.372 | -8.783 ; 6.039 | 0.715 |
| Age | -0.089 | -0.39 ; 0.213 | 0.562 |
| BDI | -1.054 | -1.153 ; -0.756 | <0.001 |
| **Multiple regression** |  |  |  |
| ALSFRS-R total | 0.523 | 0.185 ; 0.861 | <0.01 |
| ALSFRS-R bulbar | -0.74 | -1.663 ; 0.184 | 0.116 |
| BDI | -0.915 | -1.233 ; -0.596 | <0.001 |
| **Final model after backwards selection** |  |  |  |
| **ALSFRS-R total** | **0.374** | **0.09 ; 0.659** | **<0.01** |
| ALSFRS-R bulbar | -0.74 | -1.663 ; 0.184 | 0.116 |
| **BDI** | **-0.889** | **-1.208 ; -0.571** | **<0.001** |

| **Dependent Variable: Social Functioning** | | | |
| --- | --- | --- | --- |
| **Simple regression** | | | |
| **Predictor Variable** | **Regressioncoeff. β** | **95% CI for β** | **p-value** |
| ALSFRS-R total | 0.798 | 0.373 ; 1.222 | <0.001 |
| ALSFRS-R bulbar | 1.787 | 0.561 ; 3.013 | <0.01 |
| Disease duration | -0.013 | -0.126 ; 0.101 | 0.827 |
| Gender | 3.748 | -5.741 ; 13.236 | 0.436 |
| Onset | -3.616 | -14.374 ; 7.141 | 0.508 |
| Age | -0.576 | -0.989 ; -0.162 | <0.01 |
| BDI | -1.626 | -2.056 ; -1.197 | <0.001 |
| **Multiple regression** |  |  |  |
| ALSFRS-R total | 0.256 | -0.236 ; 0.748 | 0.305 |
| ALSFRS-R bulbar | 0.119 | -1.248 ; 1.486 | 0.864 |
| Age | -0.41 | -0.793 ; -0.027 | <0.05 |
| BDI | -1.458 | -1.927 ; -0.989 | <0.001 |
| **Final model after backwards selection** |  |  |  |
| ALSFRS-R total | 0.28 | -0.126 ; 0.686 | 0.175 |
| ALSFRS-R bulbar | 0.119 | -1.248 ; 1.486 | 0.864 |
| **Age** | **-0.415** | **-0.793 ; -0.037** | **<0.05** |
| **BDI** | **-1.581** | **-2.015 ; -1.148** | **<0.001** |

| **Dependent Variable: Emotional Role** | | | |
| --- | --- | --- | --- |
| **Simple regression** | | | |
| **Predictor Variable** | **Regressioncoeff. β** | **95% CI for β** | **p-value** |
| ALSFRS-R total | 0.921 | 0.163 ; 1.68 | <0.05 |
| ALSFRS-R bulbar | 1.195 | -1.023 ; 3.412 | 0.288 |
| Disease duration | 0.002 | -0.194 ; 0.198 | 0.984 |
| Gender | 3.508 | -13.315 ; 20.331 | 0.681 |
| Onset | 10.071 | -8.126 ; 28.268 | 0.276 |
| Age | -0.209 | -0.954 ; 0.536 | 0.579 |
| BDI | -2.66 | -3.392 ; -1.927 | <0.001 |
| **Multiple regression** |  |  |  |
| ALSFRS-R total | -0.105 | -0.827 ; 0.617 | 0.774 |
| BDI | -2.707 | -3.512 ; -1.902 | <0.001 |
| **Final model after backwards selection** |  |  |  |
| ALSFRS-R total | -0.105 | -0.827 ; 0.617 | 0.774 |
| **BDI** | **-2.66** | **-3.392 ; -1.927** | **<0.001** |

| **Dependent Variable: Mental Health** | | | |
| --- | --- | --- | --- |
| **Simple regression** | | | |
| **Predictor Variable** | **Regressioncoeff. β** | **95% CI for β** | **p-value** |
| ALSFRS-R total | 0.318 | -0.005 ; 0.641 | 0.053 |
| ALSFRS-R bulbar | 0.693 | -0.217 ; 1.604 | 0.134 |
| Disease duration | 0.051 | -0.031 ; 0.132 | 0.222 |
| Gender | -0.277 | -7.307 ; 6.752 | 0.938 |
| Onset | 2.319 | -5.41 ; 10.049 | 0.554 |
| Age | -0.345 | -0.66 ; -0.03 | <0.05 |
| BDI | -1.623 | -1.884 ; -1.363 | <0.001 |
| **Multiple regression** |  |  |  |
| ALSFRS-R total | -0.197 | -0.498 ; 0.103 | 0.196 |
| ALSFRS-R bulbar | -0.247 | -1.074 ; 0.579 | 0.555 |
| Age | -0.133 | -0.366 ; 0.1 | 0.26 |
| BDI | -1.691 | -1.975 ; -1.408 | <0.001 |
| **Final model after backwards selection** |  |  |  |
| ALSFRS-R total | -0.247 | -0.497 ; 0.003 | 0.052 |
| ALSFRS-R bulbar | -0.247 | -1.074 ; 0.579 | 0.555 |
| Age | -0.123 | -0.352 ; 0.107 | 0.292 |
| **BDI** | **-1.602** | **-1.865 ; -1.339** | **<0.001** |

| **Dependent Variable: BDI** | | | |
| --- | --- | --- | --- |
| **Simple regression** | | | |
| **Predictor Variable** | **Regressioncoeff. β** | **95% CI for β** | **p-value** |
| ALSFRS-R total | -0.331 | -0.462 ; -0.199 | <0.001 |
| ALSFRS-R bulbar | -0.745 | -1.128 ; -0.361 | <0.001 |
| Disease duration | -0.003 | -0.039 ; 0.033 | 0.859 |
| Gender | 1.587 | -1.492 ; 4.666 | 0.31 |
| Onset | 0.841 | -2.642 ; 4.325 | 0.634 |
| Age | 0.127 | -0.11 ; 0.266 | 0.071 |
| **Multiple regression** |  |  |  |
| ALSFRS-R total | -0.278 | -0.442 ; -0.114 | 0.001 |
| ALSFRS-R bulbar | -0.226 | -0.698 ; 0.246 | 0.345 |
| Age | 0.091 | -0.041 ; 0.222 | 0.176 |
| **Final model after backwarts selection** |  |  |  |
| **ALSFRS-R total** | **-0.331** | **-0.462 ; -0.199** | **<0.001** |
| ALSFRS-R bulbar | -0.226 | -0.698 ; 0.246 | 0.345 |
| Age | 0.101 | -0.029 ; 0.231 | 0.126 |
