# Supplementary material for: Interaction of physical function, quality of life and depression in Amyotrophic lateral sclerosis: characterization of a large patient cohort
Source: BMC Neurol. 2015 May 16;15:84. doi: 10.1186/s12883-015-0340-2 (PMC4493831; doi:10.1186/s12883-015-0340-2)
Supplement: Additional file 2: Table S2. — Association of different patient characteristics with depression (BDI without somatic items) and quality of life (SF-36) scores. [file 12883_2015_340_MOESM2_ESM.doc]

|  | **BDI** | **BDI without somatic items** | **Physical Functioning** | **Physical Role** | **Bodily Pain** | **General Health** | **Vitality** | **Social Functioning** | **Emotional Role** | **Mental Health** |
| --- | --- | --- | --- | --- | --- | --- | --- | --- | --- | --- |
| **ALSFRS-R** ↓ | ↑ **(p<0.001)** | ↑ **(p=0.002)** | ↓ **(p<0.001)** | 0.092 | - | 0.731 | ↓ **(p<0.01)** | 0.175 | 0.774 | 0.052 |
| **ALSFRS-R bulbar** ↓ | 0.345 | 0.809 | - | - | ↑ **(p<0.01)** | - | 0.116 | 0.864 | - | 0.555 |
| **Disease duration** | - | - | - | - | 0.113 | - | - | - | - | - |
| **Gender** | - | - | - | - | - | - | - | - | - | - |
| **Onset** | - | **-** | **Sig.** ↓ **for spinal onset (p<0.01)** | **Sig.** ↓ **for spinal onset ( p<0.001)** | 0.743 | - | - | - | - | - |
| **Age** ↑ | 0.126 | 0.146 | 0.072 | - | - | 0.058 | - | ↓ **(p<0.05)** | - | 0.292 |
| **BDI** ↑ | n.a. | n.a. | 0.132 | ↓ **(p<0.001)** | ↓ **(p<0.05)** | ↓ **(p<0.001)** | ↓ **(p<0.001)** | ↓ **(p<0.001)** | ↓ **(p<0.001)** | ↓ **(p<0.001)** |
| **BDI without somatic items ↑** | n.a. | n.a. | 0.099 | 0.079 | 0.99 | ↓ **(p<0.001)** | ↓ **(p<0.001)** | ↓ **(p<0.001)** | ↓ **(p<0.001)** | ↓ **(p<0.001)** |

**Additional file 2: Table S2**

**Association of different patient characteristics with depression (BDI) and quality of life (SF-36) scores**

-: p-value >0.2 in the simple regression, variable not included in the final multiple regression model

n.a.: not applicable

ALSFRS-R: Amyotrophic Lateral Sclerosis Functional Rating Scale – Revised

BDI: Beck Depression Inventory
